# Preventing disease progression in multiple sclerosis—insights from large real-world cohorts

**DOI:** 10.1186/s13073-022-01044-8

**Published:** 2022-04-20

**Authors:** Sinah Engel, Frauke Zipp

**Affiliations:** Department of Neurology, Focus Program Translational Neuroscience (FTN) and Immunotherapy (FZI), Rhine-Main Neuroscience Network (rmn2), University Medical Center of the Johannes Gutenberg University Mainz, Langenbeckstr. 1, 55131 Mainz, Germany

**Keywords:** Biomarkers, Multi-omics, Patient stratification, Personalized treatment

## Abstract

Multiple sclerosis is a chronic neuroinflammatory disease with a highly heterogeneous disease course. Preventing lasting disability requires early identification of persons at risk and novel approaches towards patient stratification for personalized treatment decisions. In this comment, we discuss the importance of large datasets of real-world cohorts in order to address this unmet need.

## Background

Multiple sclerosis (MS) is a chronic autoimmune-mediated disease affecting the central nervous system (CNS) through inflammatory and neurodegenerative processes. Although thousands of patients have benefitted from the recent introduction of highly efficient immunotherapies, including monoclonal antibodies against integrins (natalizumab), CD52 (alemtuzumab), and CD20 (ocrelizumab, ofatumumab), there is also a considerable proportion of patients who experience ongoing disease activity and progression of disability. Within 20 years of diagnosis, 30–60% of patients with initial episodic clinical attacks followed by complete or partial recovery (so-called relapsing-remitting MS (RRMS)) convert to a secondary progressive disease course (SPMS) with relapse-independent disability progression resulting in severe limitations of the quality of life, whereas others are spared from persisting neurological deficits despite long disease duration and old age [1]. This apparent heterogeneity between individual patients highlights the importance of more personalized treatment approaches, especially in order to prevent disease progression. Real-world cohorts are a key element to address this unmet need, since datasets from large observational studies, registries, and *multi-omics* approaches allow pharmaceutically independent testing of hypotheses in the presence of high heterogeneity in disease pathology, course, therapy response, and occurrence of side effects. Of similar importance are novel methodologies for prognostication and patient stratification, in particular, in deeply phenotyped (i.e., patients with available data on multiple molecular levels) real-world cohorts.

## Prevention and early diagnosis of MS

Insights gained from large international real-world cohorts demonstrate that early efficacious treatment is crucial for the long-term outcome of MS patients. However, there is increasing evidence of substantial neuronal damage already occurring in presymptomatic patients (e.g., in patients with abnormalities in magnetic resonance imaging (MRI) without clinical symptoms, the so-called radiologically isolated syndrome) and even in a prodromal phase that precedes the detection of first abnormalities in MRI. For example, increased neurofilament light chain (NfL) levels, a biomarker of neuronal damage, have been detected in blood samples obtained from seemingly healthy US military personnel up to 6 years before they presented with the first clinical episode of MS [2]. Moreover, large-scale cognitive testing of more than 20,000 individuals revealed that men later receiving a diagnosis of MS had lower cognitive scores than healthy controls up to 2 years before their first clinical event [3]. These observations highlight that delaying treatment initiation until clinical disease onset might be too late for preventing long-term progression of physical and cognitive impairment. Thus, strategies that enable the identification of individuals at risk and of patients with presymptomatic MS along with the initiation of preventive measures (e.g., modification of known risk factors like smoking and obesity, disease-specific education, and early detection examinations) might be a key to overcoming MS progression.

To date, studies by the International Multiple Sclerosis Genetics Consortium (IMSGC), an international research collaboration aiming to identify the genetic basis of MS and its disease course, have identified 233 independent genome-wide significant associations with MS susceptibility by leveraging genotype data from more than 48,000 MS patients and almost 70,000 controls [4]. Of note, many of these associations would not have been detected in smaller-scope studies due to minor contributions of individual variants to the overall genetic risk. There have already been attempts, for example, through the development of polygenic risk scores, to leverage the knowledge of these genetic risk variants in order to identify persons at risk to develop MS. In addition, the clinical implementation of new biomarkers such as serum NfL [5] may be feasible in the near future. With regard to enabling an earlier diagnosis, two recent studies conducted in large national registries provide interesting insights into how the population-wide screening of electronic health records could be used to identify MS patients before they perceive signs of neurological deficits. The first is a matched cohort study that used data linked from health administrative and clinical databases from four Canadian provinces and included 14,428 MS patients and 72,059 matched controls. The authors observed a steadily increasing annual healthcare use between 5 years and 1 year before the first demyelinating event [6]. In a second population-based observational study, the occurrence of a variety of clinical disturbances with particular attention to autonomic symptoms, psychiatric conditions, cognitive impairment, fatigue, and pain was compared between 10,204 patients who would later receive a diagnosis of MS or clinically isolated syndrome (i.e., a clinical episode suggestive of MS but not fulfilling the diagnostic criteria of dissemination in time and space) and 39,448 controls. Remarkably, MS patients had a significantly higher risk of presenting with symptoms like gastrointestinal disturbances, anxiety and mood disorders, fatigue, and pain up to 10 years prior to the first mention of an MS diagnosis in their healthcare records. The authors suggested that the integration of these symptoms into the diagnostic procedure might aid in earlier diagnosis [7].

Findings from these real-world cohort studies have considerably contributed to our understanding of MS disease course as they have led to the identification of a presymptomatic phase (the MS prodrome) and have highlighted the importance of timely preventive measures. Moreover, they indicate potential strategies for the early identification of persons at risk.

## Prognostication for personalized treatment

Large datasets and intelligent computational approaches have laid the foundation for current evidence on demographic, clinical, MRI, and cerebrospinal fluid- and blood-based biomarkers with prognostic potential in MS that may aid patient stratification and consultation. For example, in a recent international collaborative work including almost 3,000 MS patients, higher age at disease onset, reaching moderate disability levels due to persisting neurological deficits reflected by an Expanded Disability Status Scale (EDSS) score of at least 3 points within the first year after disease onset, and impairment of motor functions were associated with aggressive MS [8]. On the other hand, younger age at disease onset, lower number of relapses within the first years after onset, and full recovery from the first relapse were associated with a favorable disease course, as was being female [9]. An additional recent study aimed to define MS subtypes based on a data-driven assessment of MRI alterations [10]. By applying a novel unsupervised machine learning algorithm with the ability to model heterogeneity of phenotypes along with distinct temporal progression patterns in almost 10,000 MRI scans of patients with MS and healthy controls, the authors identified three MRI-based subtypes: cortex-led, normal-appearing white matter-led, and lesion-led. Remarkably, these subtypes differed with regard to disability progression, disease activity, and treatment response, which suggests that they reflect different pathobiological mechanisms relevant to disease manifestation and could be used for patient stratification and personalized treatment decisions [10].

Importantly, a recent genome-wide association study of MS disease progression conducted by the IMSGC was able to demonstrate for the first time that there is a genetic basis for MS severity that is clearly distinguishable from the genetic architecture of MS susceptibility [11]. This highlights a link between the molecular background of MS and its phenotypic presentation. However, genetic variation alone will not be able to unravel a condition as complex as MS, since it is most probable that hundreds of weak genetic factors interact with each other and the environment to elicit disease phenotypes. Therefore, it is necessary to also investigate data types that reflect biological processes more dynamically, e.g., in an integrative *multi-omics* approach. For example, quantitative gene expression data obtained from transcriptomic or proteomic studies could be combined with knowledge of genetic risk factors (after solving interface problems with existing databases and data harmonization), as is done in quantitative trait locus analyses that investigate the associations between risk loci and gene expression levels, in order to better understand the mechanisms involved in MS pathogenesis and for the stratification of patients into disease endotypes. To this aim, deeply phenotyped cohorts are needed.

## Conclusions

The current major challenges in the management of MS patients include the identification of persons at risk to enable preventive strategies and early diagnosis along with patient stratification for prognostication and targeted treatment decisions. Large real-world cohorts have already contributed to our understanding of pathology and disease entities as well as of treatment responses and associated adverse events. However, in order to further increase our knowledge of risk factors and to avoid persisting and progressing disability, it is necessary to foster methodological innovations that will allow us to acquire more detailed insights into the pathomechanisms, especially of the difficult-to-target local processes within the CNS. A key future task for advancing the goal of eventual translation to the clinic is the generation of large well-characterized and deeply phenotyped longitudinal datasets of patients of diverse ethnic backgrounds and diverse conditions (e.g., response to nutrition, environment, therapy) to enable sufficiently powered study designs that may yield generalizable results. In our opinion, this can only be achieved through the integration of existing real-world cohorts including biosample collections, in an international and multi-disciplinary collaboration of researchers, clinicians, and patients alike.

## Data Availability

Not applicable.

## Abbreviations

CIS: Clinically isolated syndrome
CNS: Central nervous system
EDSS: Expanded Disability Status Scale
IMSGC: International Multiple Sclerosis Genetics Consortium
MRI: Magnetic resonance imaging
MS: Multiple sclerosis
NfL: Neurofilament light chain
RRMS: Relapsing-remitting multiple sclerosis
SPMS: Secondary progressive multiple sclerosis
